# Association of Preoperative Serum Levels of CEA and CA15-3 with Molecular Subtypes of Breast Cancer

**DOI:** 10.1155/2021/5529106

**Published:** 2021-09-27

**Authors:** Wenjing Zhao, Xiaoyan Li, Wenqing Wang, Bing Chen, Lijuan Wang, Ning Zhang, Zhe Wang, Qifeng Yang

**Affiliations:** ^1^Pathology Tissue Bank, Qilu Hospital of Shandong University, Jinan, Shandong, China; ^2^Department of Breast Surgery, General Surgery, Qilu Hospital of Shandong University, Jinan, Shandong, China; ^3^Research Institute of Breast Cancer, Shandong University, China

## Abstract

**Objectives:**

Molecular subtypes are employed as a guide for targeted treatment and important prognostic factors. This study focused on investigating the association of serum levels of CEA, CA15-3, and CA125 with clinicopathological characteristics of breast cancer to find prognostic markers for breast cancer and provide precise targeted therapy.

**Materials and Methods:**

In this study, 961 breast cancer patients with preoperative serum levels of CEA, CA15-3, and CA125 and molecular subtypes were analyzed. Cut-off values of 5 ng/ml, 25 U/ml, and 35 U/ml were used for CEA, CA15-3, and CA125, respectively. The *χ*^2^ test and Fisher exact test along with logistic multivariate regression analysis were performed for investigating the correlation of CEA, CA15-3, and CA125 serum levels with molecular subtypes and associated factors.

**Results:**

An increase in the serum concentrations of CEA, CA15-3, and CA125 was discovered in 48 (4.99%), 54 (5.62%), and 55 (5.72%) breast cancer patients, respectively. Univariate analysis demonstrated that the levels of CEA (*p* < 0.01) and CA15-3 (*p* < 0.05) were significantly linked with molecular types of breast cancer. Moreover, patients having larger tumor size (*p* < 0.01, *p* < 0.0001, and *p* < 0.05, respectively) along with nodal metastasis (*p* < 0.05, *p* = 0.0001, and *p* < 0.05, respectively) exhibited higher rates of elevated CEA, CA15-3, and CA125 levels. Status of Her-2 positive (*p* < 0.01) had a significant connection with elevated CEA levels. Multivariate analysis further indicated that molecular subtypes were independent factors associated with CEA and CA15-3 levels. Also, Her-2 status was significantly and independently related to CEA levels.

**Conclusion:**

Preoperative serum levels of CEA and CA15-3 were independently associated with molecular subtypes of breast cancer. CEA and CA15-3 might improve the prognostic prediction for patients with breast cancer and inform the selection of specific therapies. A further biological analysis is needed for investigating the relationship between Her-2 expression and CEA levels.

## 1. Introduction

Female breast cancer has now surpassed lung cancer as the leading cause of global cancer incidence in 2020, with an estimated 2.3 million new cases, representing 11.7% of all cancer cases [1]. In women, breast cancer is the most commonly diagnosed cancer and the leading cause [1]. In China, rates of breast cancer incidence and mortality have been accelerating over years and which were projected to increase modestly in the future [2]. Although there is a surge in the incidence of breast cancer, however, timely detection and the use of effective systemic adjuvant therapy while following prognostic factors have improved breast cancer prognosis [3, 4]. Cancer antigen 15-3 (CA15-3) in addition to carcinoembryonic antigen (CEA) along with cancer antigen 125 (CA125) are extensively used in the clinical practice of breast cancer as serum tumor markers. These have been developed as noninvasive, easily available, and cost-effective tumor markers for immediate diagnosis, monitoring, and prediction of breast cancer [5–7].

Breast cancer is documented as a heterogeneous disease that is further divided into molecular subtypes based on different genetic, molecular, and pathological characteristics [8]. Furthermore, breast cancer has been categorized into five molecular subtypes based on immunohistochemical factors which include progesterone receptor (PR), estrogen receptor (ER), Ki67 proliferation index, and human epidermal growth factor receptor 2 (Her-2). Furthermore, these five molecular subtypes are named luminal A in addition to luminal B (Her-2 positive) along with luminal B (Her-2 negative) apart from Her-2 overexpression as well as triple-negative [9]. Similarly, molecular subtypes along with the four immunohistochemical factors are employed as a guide for targeted treatment, including hormonal therapy, anti-Her2 agent, and cytotoxic therapy [8, 10, 11]. Moreover, differences in prognosis among different subtypes of breast cancer also existed [12–14].

Further investigation is needed to identify the clinical significance of tumor markers encompassing CA125, CA15-3, and CEA. Presently, the association of molecular subtypes of breast cancer with preoperative serum levels of CA125, CA15-3, and CEA has not been elucidated yet. Therefore, this study focused on investigating the correlation of tumor markers encompassing CEA, CA15-3, and CA125 levels with molecular subtypes to improve the prognostic prediction for breast cancer and inform the appropriate therapy.

## 2. Materials and Methods

### 2.1. Study Patients

From January 2014 to December 2017, preoperative serum CEA, CA15-3, and CA125 concentration levels from overall 961 patients who received treatment at Qilu Hospital of Shandong University (Shandong, Jinan) were retrospectively investigated. Each recruited patient had stage I-III invasive breast cancer. Moreover, patients who had stage IV carcinoma, concurrent bilateral breast cancer, unknown histological, and tumor marker information were excluded.

### 2.2. Measurement of CEA, CA15-3, and CA125 Levels and Immunohistochemical Factors

Before initiating surgery, 5 ml of peripheral blood was sampled from each recruited patient. Similarly, we employed an automatic electrochemiluminescence immunoassay system (Roche E601, Germany) for measuring preoperative serum levels of CEA, CA15-3, and CA125. Cut-off values of 5 ng/ml, 25 U/ml, and 35 U/ml were used, respectively, for CEA, CA15-3, and CA125 as suggested levels by the manufacturer.

Positivity for ER and PR was determined when tumors with at least 1 percent of nuclear stained cells. Furthermore, we determined Her-2 positivity by 3+ or 2+ score while passing through immunohistochemical evaluation, which was then passed through in situ hybridization (FISH) for further confirmation. Moreover, for Ki-67 measurement, a threshold value of 14 percent was used to distinguish tumors with low and high proliferation. According to immunohistochemical factors, molecular subtypes of breast cancer were classified as given next: First of all, luminal A consists of either both ER and PR positive or only one of them in addition to HER-2 negative along with Ki − 67 < 14%, while luminal B1 consists of either both ER and PR positive or only one of them in addition to HER-2 negative along with Ki − 67 ≥ 14%. Similarly, luminal B2 consists of either both ER and PR positive or only one of them in addition to HER-2 positive. Moreover, the fourth type which is HER-2 overexpression consists of both ER and PR negative in addition to HER-2 positive. The last and fifth type is triple-negative wherein all three are negative including ER, PR, and HER-2.

### 2.3. Statistical Analysis

SAS 9.4 statistical software package was used to analyze all the data. The correlation between CEA, CA125, and CA15-3 levels and immunohistochemical factors and other associated factors was examined by univariate analysis while employing the *χ*^2^ test and Fisher exact test. Moreover, stepwise enrollment of significant variables from univariate analysis into that of multivariate analysis was carried out. Similarly, logistic regression analysis was used for identifying independent factors correlated with the levels of CA15-3, CEA, and CA125. Two-side *p* values were reported in this study. *p* values that were less than 0.05 were regarded as significant.

## 3. Results

### 3.1. Patient Characteristics and Clinicopathological Status

Table 1 shows the clinicopathological status of all the patients enrolled in this study. The population of the study had a mean age of 51.00 ± 10.72 years. High levels of serum (exceeding the cut-off values) of CEA, CA15-3, and CA125 were determined in 48 (4.99%), 54 (5.62%), and 55 (5.72%) breast cancer patients, respectively. Analysis of molecular subtypes of breast cancer indicated that 23.31% of patients had luminal A subtype, 39.96% had luminal B1 (Her-2 negative) subtype, 13.22% had luminal B2 (Her-2 positive) subtype, 11.34% had Her-2 overexpression subtype, and 12.17% had triple-negative subtype.

### 3.2. Univariate Analysis of Association between CEA, CA15-3, and CA125 and Molecular Subtypes of Breast Cancer and Clinicopathological Factors

Table 2 shows the association of serum levels of CEA, CA15-3, and CA125 with the molecular subtypes of breast cancer. It was revealed by the univariate analysis that levels of CEA level (*p* < 0.01) and CA15-3 (*p* < 0.05) had a significant association with molecular subtypes of breast cancer; however, no such significant association was found for CA125 level. Similarly, Her-2 overexpression and luminal B2 (Her-2 positive) subtypes demonstrated higher rates of elevated CEA level. Meanwhile, triple-negative and luminal B2 (Her-2 positive) subtypes had a higher probability to exhibit elevation in CA15-3 level.

Tables 3, 4, and 5 show the results of the univariate analysis of the association between serum CEA, CA15-3, and CA125 levels and clinicopathological factors. Furthermore, tumor size (*p* < 0.01, *p* < 0.0001, and *p* < 0.05, respectively) showed a significant association with a rise in the levels of CEA, CA15-3, and CA125. Similarly, patients with nodal metastasis (*p* < 0.05, *p* = 0.0001, and *p* < 0.05, respectively) had a higher probability of elevation in the levels of CEA, CA15-3, and CA125. Additionally, the negative status of PR (*p* < 0.01 and *p* < 0.05, respectively) had a significant association with serum levels of CA15-3 and CA125. Furthermore, patients with breast cancer aged 50 years and above (*p* < 0.05) and with the status of Her-2 positive (*p* < 0.01) exhibited higher rates of elevated CEA level. Also, patients with a negative status of ER and higher histological grade tend to have an elevated level of CA15-3. Moreover, the Ki67 value was not significantly associated with all three tumor markers.

### 3.3. Multivariate Analysis of the Association between CEA, CA15-3, and CA125 and Molecular Subtypes of Breast Cancer and Clinicopathological Factors

Multivariate logistic regression analysis of the association between the serum levels of CA15-3, CEA, and CA125 and related factors is shown in Table 6. Model 1 included age, tumor size, and molecular type, while in model 2, age, tumor size, and Her-2 status were included. Analysis of these two models showed that age, tumor size, molecular type, and Her-2 status were independent factors associated with CEA level. As a reference to the Her-2 overexpression subtype, luminal B1 exhibited a lower risk of elevated CEA level (OR = 0.386, 95%CI = 0.163 − 0.918, *p* < 0.05). Moreover, patients with Her-2 positive status were more likely to have an elevated CEA level.

Model 3 incorporated histologic grade, tumor size, node status, and molecular type. Model 4 added ER and PR status, while the molecular type was excluded. Results of models 3 and 4 indicated that the size of the tumor along with node status and molecular type was fundamentally and independently related to the CA15-3 level. Compared to the luminal B2 subtype, luminal B1 exhibited a lower risk of having a higher CA15-3 level.

Model 5 included node status, tumor size, and PR status, which identified that both node status and PR status were independent factors of having an elevated CA125 level.

## 4. Discussion

In our present study, 961 patients carrying an invasive form of breast cancer were recruited to investigate the clinical importance of identifying the preoperative serum levels of various tumor markers such as CEA, CA15-3, and CA125. This study also focused on investigating the association between levels of CEA, CA15-3, and CA125 and different molecular subtypes of breast cancer. Therefore, it was unveiled by the results that high serum levels of both CEA and CA15-3 were independently and substantially related to molecular subtypes of breast cancer.

Our study hypothesis, patient characteristics, and statistical analysis methods were referring to the guideline of reporting recommendations for tumor marker prognostic studies(REMARK) [15]. There are limited studies available on the association of CEA, CA15-3, and CA125 levels with that of the molecular subtypes of breast cancer. Wu et al. [16] reported that patients with the Her-2 overexpression subtype had a higher level of CEA than the other 3 subtypes, while in the study of Li et al. [17], luminal A subtype presents a significant high level of CEA. There was no significant difference of CA15-3 level between molecular subtypes in these two studies [16, 17]. The results of the other two studies [18, 19] unveiled that levels of CEA and CA15-3 were not significantly related to molecular subtypes. In our current study, luminal B2 (ER and/or PR positive, Her-2 positive) and Her-2 overexpression subtypes demonstrated higher rates of elevated CEA level, and triple-negative subtype exhibited a lower level of elevated CEA, which were in harmony with the study of Wu et al. [16]. Also, we discovered that a significantly higher CA15-3 level was seen in luminal B2 and triple-negative subtypes. In our study, we further identified molecular subtype retained its significant association with CEA and CA15-3 levels even when adjustments were made for other known related clinicopathological factors. Serum level of CA125 was not found to be significantly different in molecular subtypes. Previous studies have shown that elevated level of CEA was correlated to Her-2 positive status [18, 20, 21]. Besides, we also observed a significant difference of higher CEA level in Her-2 status but not in ER, PR status, and Ki67 value. The difference of elevated CEA level between molecular types may chiefly be caused by the significant correlation between higher CEA levels and Her-2 status. Her-2 amplification had been identified in human breast cancer cells which may result in a more aggressive tumor type clinically and significantly related to survival in patients with breast cancer [22–26]. Her-2 and serum CEA level are both widely used markers for the prognosis of breast cancer. The biological behavior between elevated CEA level and Her-2 overexpression needs to be further explored.

Previous studies have already shown the association between tumor size, which represents the tumor burden, and elevated levels of CEA or CA15-3 [16, 17, 21, 27]. In the present study, we also identified the association between tumor size and elevation in the levels of CEA, CA15-3, and CA125. Meanwhile, tumor size was further identified as an independent factor associated with CEA, CA15-3, and CA125 levels in the multivariate analysis. Significantly high levels of CEA or CA 15-3 were exhibited in patients suffering from a later stage of breast cancer than those with early disease [17, 28]. In agreement with previous reports [21, 29, 30], we also demonstrated that elevated preoperative serum level of CA15-3 was related to poor histological grade in breast cancer. However, the relationship between tumor biological index and CEA, CA15-3, and CA125 levels is not clearly confirmed yet [6, 31].

In our present study, we identified that patients carrying high serum levels of CEA and CA15-3 were more likely to exhibit metastasis status of lymph node, which was in harmony with the studies of Wu et al. [16, 17, 32]. Lee et al. [21] also reported the relation between CA15-3 level and lymph node metastasis status. Our study also indicated that CA125 was significantly associated with node status and CA15-3 and CA125 were independent factors related to node status. Cancer antigen in serum implies the possibility of tumor vascularization and micrometastases [33]. Also, Kim et al. measured the concentration of CA15-3 in washout of fine-needle aspirates (FNA) from the axillary lymph node of patients having breast cancer, identifying that patients diagnosed with positive metastasis had remarkably elevated FNA concentrations of CA-15-3 as compared to patients without lymph node metastasis [34]. This may partly explain the positive relationship between elevated CA15-3 and CA125 levels and metastasis status of the lymph node. The biological mechanism of lymph node and CA15-3 and CA125 levels needs to be further investigated.

There were a few limitations to the present study. First of all, this was a single-center retrospective analysis with bias in subject selection. Furthermore, only qualitative analysis of serum levels was focused for CEA, CA15-3, and CA125. Therefore, further prospective investigation in addition to quantitative analysis of CEA, CA15-3, and CA125 levels is needed to be carried out.

## 5. Conclusion

In conclusion, the study demonstrated that preoperative serum levels of CEA and CA15-3 had an independent association with molecular subtypes of breast cancer with high level of CEA in Her-2-positive patients (luminal B Her-2 positive and Her-2 overexpression). The study also explored the indicated significance of serum tumor markers in larger tumor size, Her-2 overexpression, metastasis status of lymph node, older age, and poor histological grade. Further biological analysis and prospective study are needed to be carried out for Her-2 expression and serum levels of CEA.

## Figures and Tables

**Table 1:** General characteristics of study population.

Characteristics	*N*	Percentage (%)
All	961	
Age (years)		
<50	454	47.24
≥50	507	52.76
Histologic grade		
Grade 1	70	8.10
Grade 2	589	68.17
Grade 3	205	23.73
Tumor size (cm)		
<2	516	57.40
≥2	383	42.60
Node status		
N0	508	55.52
N1	407	44.48
ER		
Negative	251	26.12
Positive	710	73.78
PR		
Negative	292	30.42
Positive	669	69.58
Her-2		
Negative	725	75.44
Positive	236	24.56
Ki67 (%)		
<14	257	26.80
≥14	702	73.20
Molecular types		
Luminal A	224	23.31
Luminal B1	384	39.96
Luminal B2	127	13.22
Her-2+	109	11.34
Triple-negative	117	12.17
CEA (ng/ml)		
<5	913	95.01
≥5	48	4.99
CA153 (U/ml)		
<25	907	94.38
≥25	54	5.62
CA125 (U/ml)		
<35	906	94.28
≥35	55	5.72

Luminal B1: ER+/PR+, Her-2 negative; Luminal B2: ER+/PR+, Her-2 positive; Her-2+: Her-2 overexpression.

**Table 2:** Correlation between serum CEA, CA15-3, and CA125 levels and molecular subtypes in breast cancer.

Tumor marker	Luminal A (*n*(%))	Luminal B1 (*n*(%))	Luminal B2 (*n*(%))	Her-2+ (*n*(%))	Triple-negative (*n*(%))	*p* value
CEA (ng/ml)						0.0194^∗^
<5	213 (95.09)	371 (96.61)	117 (92.13)	98 (89.91)	114 (97.44)	
≥5	11 (4.91)	13 (3.39)	10 (7.87)	11 (10.09)	3 (2.56)	
CA15-3 (U/ml)						0.0007^∗∗∗^
<25	216 (96.43)	371 (96.61)	114 (89.76)	103 (94.50)	103 (88.03)	
≥25	8 (3.57)	13 (3.39)	13 (10.24)	6 (5.50)	14 (11.97)	
CA125 (U/ml)						0.2605
<35	213 (95.09)	363 (94.53)	121 (95.28)	104 (95.41)	105 (89.74)	
≥35	11 (4.91)	21 (5.47)	6 (4.72)	5 (4.59)	12 (10.26)	

Luminal B1: ER+/PR+, Her-2 negative; Luminal B2: ER+/PR+, Her-2 positive; Her-2+: Her-2 overexpression. ^∗^*p* < 0.05 and ^∗∗∗^*p* < 0.001.

**Table 3:** Correlation between serum CEA level and clinicopathological factors.

Characteristic	CEA negative (*n*(%))	CEA positive (*n*(%))	*p* value
Age (years)			<0.0001^∗∗∗^
<50	445 (98.02)	9 (1.98)	
≥50	468 (92.31)	39 (7.69)	
Histologic grade			0.9583
Grade 1	67 (95.71)	3 (4.29)	
Grade 2	563 (95.59)	26 (4.41)	
Grade 3	195 (95.12)	10 (4.88)	
Tumor size (cm)			0.0031^∗∗^
<2	502 (97.29)	14 (2.71)	
≥2	356 (92.95)	27 (7.05)	
Node status			0.0446^∗^
N0	491 (96.65)	17 (3.35)	
N1	382 (93.86)	25 (6.14)	
ER			0.4064
Negative	236 (94.02)	15 (5.98)	
Positive	677 (95.35)	33 (4.65)	
PR			0.2647
Negative	274 (93.84)	18 (6.16)	
Positive	639 (95.52)	30 (4.48)	
Her-2			0.0030^∗∗^
Negative	698 (96.28)	27 (3.72)	
Positive	215 (91.10)	21 (8.90)	
Ki67 (%)			0.8681
<14	245 (95.33)	12 (4.67)	
≥14	666 (94.87)	36 (5.13)	

^∗^*p* < 0.05, ^∗∗^*p* < 0.01, and ^∗∗∗^*p* < 0.001.

**Table 4:** Correlation between serum CA15-3 level and clinicopathological factors.

Characteristic	CA15-3 negative (*n*(%))	CA15-3 positive (*n*(%))	*p* value
Age (years)			0.4001
<50	432 (95.15)	22 (4.85)	
≥50	475 (93.69)	32 (6.31)	
Histologic grade			0.0223^∗^
Grade 1	69 (98.57)	1 (1.43)	
Grade 2	562 (95.42)	27 (4.58)	
Grade 3	187 (91.22)	18 (8.78)	
Tumor size (cm)			<0.0001^∗∗∗^
<2	503 (97.48)	13 (2.52)	
≥2	348 (90.86)	35 (9.14)	
Node status			0.0001^∗∗∗^
N0	493 (97.05)	15 (2.95)	
N1	371 (91.15)	36 (8.85)	
ER			0.0036^∗∗^
Negative	227 (90.44)	24 (9.56)	
Positive	680 (95.77)	30 (4.23)	
PR			0.0056^∗∗^
Negative	266 (91.10)	26 (8.90)	
Positive	641 (95.81)	28 (4.19)	
Her2			0.0729
Negative	690 (95.17)	35 (4.83)	
Positive	217 (91.95)	19 (8.05)	
Ki67 (%)			0.2050
<14	247 (96.11)	10 (3.89)	
≥14	658 (93.73)	44 (6.27)	

^∗^*p* < 0.05, ^∗∗^*p* < 0.01, and ^∗∗∗^*p* < 0.001.

**Table 5:** Correlation between serum CA125 level and clinicopathological factors.

Characteristic	CA125 negative (*n*(%))	CA125 positive (*n*(%))	*p* value
Age (years)			0.1674
<50	423 (93.17)	31 (6.83)	
≥50	483 (95.27)	24 (4.73)	
Histologic grade			0.6247
Grade 1	65 (92.86)	5 (7.14)	
Grade 2	558 (94.74)	31 (5.26)	
Grade 3	191 (93.17)	14 (6.83)	
Tumor size (cm)			0.0296^∗^
<2	494 (95.74)	22 (4.26)	
≥2	353 (92.17)	30 (7.83)	
Node status			0.0157^∗^
N0	487 (95.87)	21 (4.13)	
N1	374 (91.89)	33 (8.11)	
ER			0.0825
Negative	231 (92.03)	20 (7.97)	
Positive	675 (95.07)	35 (4.93)	
PR			0.0341^∗^
Negative	268 (91.78)	24 (8.22)	
Positive	638 (95.37)	31 (4.63)	
Her2			0.5191
Negative	681 (93.93)	44 (6.07)	
Positive	225 (95.34)	11 (4.66)	
Ki67 (%)			
<14	244 (94.94)	13 (5.06)	0.5855
≥14	660 (94.02)	42 (5.98)	
Molecular subtype			0.2605
Luminal A	213 (95.09)	11 (4.91)	
Luminal B1	363 (94.53)	21 (5.47)	
Luminal B2	121 (95.28)	6 (4.72)	
Her2 overexpression	104 (95.41)	5 (4.59)	
Triple-negative	105 (89.74)	12 (10.26)	

^∗^*p* < 0.05.

**Table 6:** Multiple logistic regression analysis of the association of clinicopathological characteristics with serum CEA, CA15-3, and CA125 levels.

Characteristic	OR	CI	*p* value
Model 1 CEA			
Age (years)			
≥50 vs. <50	4.319	2.040-9.143	0.0001^∗∗∗^
Tumor size (cm)			
≥2 vs. <2	2.364	1.185-4.715	0.0146^∗^
Node status			
N1 vs. N0	1.770	0.918-3.413	0.0885
Molecular type			
Luminal A vs. Her2+	0.679	0.273-1.689	0.4056
Luminal B1 vs. Her2+	0.386	0.163-0.918	0.0313^∗^
Luminal B2 vs. Her2+	0.933	0.365-2.382	0.8840
Triple-negative vs. Her2+	0.294	0.078-1.110	0.0710
Model 2 CEA			
Age (years)			
≥50 vs. <50	4.299	2.036-9.079	0.0001^∗∗∗^
Tumor size (cm)			
≥2 vs. <2	2.262	1.141-4.482	0.0193^∗^
Node status			
N1 vs. N0	1.749	0.910-3.359	0.0934
Her2			
Positive vs. negative	2.156	1.169-3.979	0.0139^∗^
Model 3 CA15-3			
Histologic grade			
Grade 2 vs. Grade 1	1.717	0.213-13.862	0.6120
Grade 3 vs. Grade 1	2.209	0.228-18.062	0.5260
Tumor size (cm)			
≥2 vs. <2	3.019	1.538-5.926	0.0013^∗∗^
Node status			
N1 vs. N0	2.633	1.387-5.001	0.0031^∗∗^
Molecular type			
Luminal A vs. luminal B2	0.457	0.173-1.203	0.1127
Luminal B1 vs. luminal B2	0.349	0.154-0.791	0.0117^∗^
Her2+ vs. luminal B2	0.478	0.170-1.346	0.1620
Triple-negative vs. luminal B2	1.293	0.536-3.119	0.5676
Model 4 CA15-3			
Histologic grade			
Grade 2 vs. Grade 1	1.765	0.229-13.586	0.5851
Grade 3 vs. Grade 1	2.244	0.271-18.593	0.4536
Tumor size (cm)			
≥2 vs. <2	2.865	1.464-5.608	0.0021^∗∗^
Node status			
N1 vs. N0	2.720	1.440-5.140	0.0020^∗∗^
ER			
Positive vs. negative	0.607	0.242-1.523	0.2874
PR			
Positive vs. negative	0.794	0.327-1.924	0.6088
Model 5 CA125			
Tumor size (cm)			
≥2 vs. <2	1.550	0.863-2.782	0.1423
Node status			
N1 vs. N0	1.903	1.070-3.386	0.0286^∗^
PR			
Positive vs. negative	0.567	0.323-0.996	0.0486^∗^

^∗^*p* < 0.05, ^∗∗^*p* < 0.01, and ^∗∗∗^*p* < 0.001.

## Data Availability

All data needed to evaluate the conclusions in the paper are present in the paper.
